# An initial assessment of spatial relationships between respiratory cases, soil metal content, air quality and deprivation indicators in Glasgow, Scotland, UK: relevance to the environmental justice agenda

**DOI:** 10.1007/s10653-013-9565-4

**Published:** 2013-11-08

**Authors:** S. Morrison, F. M. Fordyce, E. Marian Scott

**Affiliations:** 1 School of Mathematics and Statistics, University of Glasgow, 15 University Gardens, Glasgow, G12 8QW Scotland, UK; 2 British Geological Survey, West Mains Road, Edinburgh, EH9 3LA Scotland, UK

**Keywords:** Soil metals, Air quality, Pollutants, Health, Deprivation, Environmental justice

## Abstract

There is growing interest in links between poor health and socio-environmental inequalities (e.g. inferior housing, crime and industrial emissions) under the environmental justice agenda. The current project assessed associations between soil metal content, air pollution (NO_2_/PM_10_) and deprivation and health (respiratory case incidence) across Glasgow. This is the first time that both chemical land quality and air pollution have been assessed citywide in the context of deprivation and health for a major UK conurbation. Based on the dataset ‘averages’ for intermediate geography areas, generalised linear modelling of respiratory cases showed significant associations with overall soil metal concentration (*p* = 0.0367) and with deprivation (*p* < 0.0448). Of the individual soil metals, only nickel showed a significant relationship with respiratory cases (*p* = 0.0056). Whilst these associations could simply represent concordant lower soil metal concentrations and fewer respiratory cases in the rural versus the urban environment, they are interesting given (1) possible contributions from soil to air particulate loading and (2) known associations between airborne metals like nickel and health. This study also demonstrated a statistically significant correlation (−0.213; *p* < 0.05) between soil metal concentration and deprivation across Glasgow. This highlights the fact that despite numerous regeneration programmes, the legacy of environmental pollution remains in post-industrial areas of Glasgow many decades after heavy industry has declined. Further epidemiological investigations would be required to determine whether there are any causal links between soil quality and population health/well-being. However, the results of this study suggest that poor soil quality warrants greater consideration in future health and socio-environmental inequality assessments.

## Introduction

Many studies have shown that populations exposed to high concentrations of potentially harmful elements (PHE) such as As, Cr, Cu, Ni, Pb, Se and Zn in the environment can have their health adversely affected (Mielke et al. 2011; Nriagu 2011; Selinus 2005; Skinner and Berger 2003; WHO 1996). Although these metals/metalloids (hereafter metals) occur naturally in soil, concentrations can be elevated as a result of anthropogenic activities such as industrialisation, transportation and waste disposal, particularly in urban environments (e.g. Birke and Rauch 2000; Fordyce et al. 2005; Johnson et al. 2011; Wong 1996). High concentrations of these metals in soil can cause health problems in some cases if high-level exposure occurs over long periods of time. For example, Chiang et al. (2011) reported associations between incidences of oral cancer in populations exposed to high soil Cr and Ni associated with electroplating industries in Taiwan. Mielke et al. (2005) also demonstrated a strong inverse association between metals in soil/dust in elementary schools in New Orleans and the educational achievement of school children, with high blood–Pb concentrations linked to learning and behavioural difficulties. Studies have also demonstrated associations between seasonal variability in blood–Pb levels in children and the re-suspension of urban soils into the atmosphere (e.g. Laidlaw and Filippelli 2008; Zahran et al. 2013). Concerns have also been expressed about the concentrations of metals (particularly As, Cd, Hg, Ni and Pb) and potential childhood exposure in urban day care centre soils in Norway, leading to a nationwide remediation programme (Ottesen et al. 2008). However, some other studies demonstrate no adverse health effects from contaminated land (RCEP 1996). Associations between terrestrial pollution and health in Western societies are often complex and causal links are hard to establish. The health impacts of some metals and the combinations of metals are yet to be fully understood (Selinus and Frank 2000).

However, the links between air pollution and health have been well established (DEFRA 2010; Dockery and Pope 1994; Patel et al. 2009), and UK guidelines have been set to improve air quality in order to protect health (UK Air Quality Archive 2007). Air quality has improved greatly since the introduction of the UK Clean Air Acts in 1956 and 1968; however, exposure to high levels of air pollution can still lead to irritation of the lungs, attacks for asthmatics and increased risks for those with lung or heart problems (DEFRA 2010).

The concept of environmental justice developed in the United States in the 1980s. It aims to remedy unequal distributions of socio-environmental problems such as poor housing, poor air quality, pollution and access to services for all communities (Bullard 2005). In recent years, there has been growing interest in socio-environmental inequalities and impacts on health in Europe since rights ‘to live in an environment adequate to a person’s health and well-being’ were incorporated in the 1998 Aarhus pan-European convention on the environment (ESRC-GECP 2001; WHO 2010). Several studies have now demonstrated links between socio-environmental problems such as poor-quality housing, crime, litter, poor air quality, proximity to pollution sources and deprivation in the United Kingdom under initiatives such as the environmental justice agenda (e.g. ESRC-GECP 2001; FoE 2001; Walker et al. 2003). In Scotland, previous work under the environmental justice agenda explored the potential health impacts within neighbourhoods of eight environmental factors such as industrial emissions, derelict land, landfill, quarries, woodlands, green space, river water quality and air quality (Scottish Government 2005). These issues were analysed in conjunction with the Scottish Index of Multiple Deprivation (SIMD 2010). Results reported by Fairburn et al. (2005) demonstrated associations between socially deprived areas and air pollution, derelict land and river water quality. However, chemical land quality was not assessed and investigations into links with pollution were preliminary (Scottish Government 2005). Similar studies have been carried out in other parts of the United Kingdom using green space and the agricultural/nature conservation value of land as land quality indicators (e.g. Midgely et al. 2005; TEP 2007), but none of these studies included the chemical quality of land.

To examine the relationships between chemical land quality and deprivation indicators, Glasgow was selected for the present study as it has a long history of urbanisation and industrialisation resulting in increased concentrations of metals in the soil. These have been mapped by the British Geological Survey (BGS) showing soil metal concentrations are elevated in the city up to three times that of rural soil in the area (Fordyce et al. 2005, 2012). Of the fourteen UK cities studied, Glasgow was reported to have the highest median soil Cr concentration (Fordyce et al. 2005). This was in large part due to the history of metal processing in the city. The world’s largest chromite ore processing works were located in south-east Glasgow from 1830 to 1968. In the past, waste from the works was used as fill material around the city, leading to concerns about potential health impacts on the local population (Farmer and Jarvis 2009). However, previous investigations found no evidence of adverse health effects (Eizaguirre-Garcia et al. 1999; Watt et al. 1991) and in recent years, the Cr-contaminated sites are being capped and remediated to reduce exposure to airborne dusts (Farmer and Jarvis 2009). Nonetheless, the BGS soil dataset provides an opportunity to test whether the poor chemical quality of land is spatially coincident with indicators of poor health and deprivation in the largest city in Scotland. This is the first time that chemical land quality, air quality, deprivation and health datasets have been combined for a major UK city. However, the purpose of this investigation was not to prove links between particular soil metals or air pollutants and specific health problems or to carry out an epidemiological study, rather to consider spatial associations and inequalities in the context of the environmental justice agenda.

## Materials and methods

### Data presentation

**Fig. 1:**
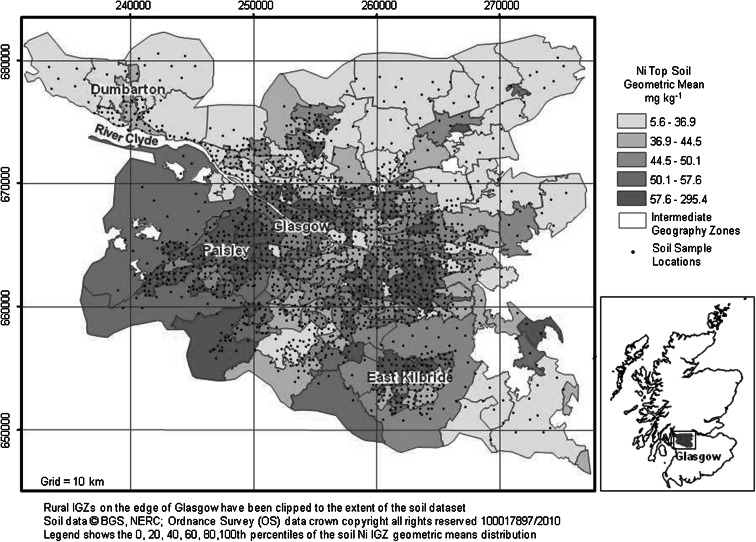
Intermediate geography zones (IGZs) (SNS 2010); soil sample locations and IGZ geometric mean top soil Ni concentrations (derived from Fordyce et al. 2012) across the Glasgow study area

To examine the relationships between the datasets used in this study, the data were required to have a common spatial scale. Since the study was dealing with health data, it was difficult to obtain data at small geographical scales due to confidentiality issues. Therefore, the smallest geographical scale for which health data were routinely available was the intermediate geography zone (IGZ). The IGZs were devised by the Scottish Government to present statistical data; they approximate to parliamentary constituencies and local authority boundaries but each IGZ includes c.4000 households of similar social characteristics (Scottish Government 2011) (Fig. 1). All map generation and geostatistical processing were carried out in ArcGIS 9.2 Geographical Information System (GIS) software (Environmental Systems Research Institute, ESRI^®^).

### Chemical land quality data

Chemical land quality information was provided by the BGS Geochemical Baseline Survey of the Environment (G-BASE) soil geochemistry dataset for Glasgow. The G-BASE soil sampling of Greater Glasgow (including Paisley, East Kilbride and Dumbarton) was carried out between 2001 and 2002, whereby 1,381 urban soil samples were collected on a systematic grid at a density of 4 per km^2^, whilst 241 rural soil samples were collected from the outskirts of Glasgow from every second km^2^. A hand-held Dutch auger was used to collect two separate soil samples at each site; a top soil (5–20 cm) and a deeper soil (35–50 cm) sample each comprising a composite from the corners and centre of a 20 × 20 m square (Fordyce et al. 2012; Johnson 2005). The top soil starting depth is recorded as 5 cm to take account of any non-soil vegetative/grass roots, which often constitute the first few centimetres of the soil profile and have to be removed. The G-BASE top soil sample nonetheless comprises the top-most soil material in the profile. The top soil data only were used in this study as the public are more likely to come into contact with surface soil rather than deeper soil whether via hand-to-mouth contact in children; airborne re-suspension of soil particulates or consumption of home-grown vegetables. Samples were air- and oven-dried at <30 °C, sieved to <2 mm, ground and analysed by X-ray Fluorescence Spectrometry (XRFS) (Fordyce et al. 2012). The subset of G-BASE soil data used for the present project comprised spatially registered total element concentrations of As, Cr, Cu, Ni, Pb, Se and Zn and K (Table 1). Potassium was included as a generally non-harmful control element. The other elements were chosen as they were deemed of potential concern to human health under the current UK Contaminated Land Exposure Assessment (CLEA) soil quality guidelines (EA 2009) or the former UK soil trigger values (ICRCL 1987). Although the CLEA guidelines also include soil Cd and Hg, Hg is not analysed in the G-BASE survey and the majority of Cd data were below the detection limit, hence could not be incorporated into the study. Exploratory data analysis revealed the soil metals data were highly positively skewed; therefore, geometric means of the data were taken as an average measure. Since a considerable departure from symmetry can cause difficulties in statistical assessments, the data were log-transformed for the purposes of the further geostatistical analysis carried out as part of the project (Morrison 2011). The soil data were in the form of point locations and were available for 279 IGZs in the Glasgow area (Fig. 1). To compare the soil information with the other datasets, the data were aggregated by taking the raw untransformed geometric mean concentrations of each metal for each IGZ. However, as Fig. 1 shows, some of the rural IGZs around Glasgow contained only a few soil samples and this resulted in greater uncertainty in the summary measures in the peripheral areas. The large rural IGZs around Glasgow were clipped to the spatial extent of the G-BASE soil dataset for presentation purposes and to avoid averaging into areas of no data.

**Table 1:** Summary statistics for Glasgow top soil metal concentrations (from Fordyce et al. 2012)

Metal (mg kg^−1^)	Mean	SD	Min	Median	Max
As	10.8	10.6	1.1	9.1	282.8
Cr	121.6	130.3	28.5	107.0	4286.0
Cu	70.5	120.0	2.9	47.8	3679.9
Ni	52.6	43.8	2.3	45.7	1038.1
Pb	167.9	210.5	13.4	118.3	5001.0
Se	1.0	0.7	0.1	0.9	14.5
Zn	189.4	175.4	13.7	144.4	1780.8
K (wt%)	1.4	0.3	0.3	1.3	3.1

*SD* Standard deviation, *Min* Minimum, *Max* Maximum

In addition to the individual concentrations for each metal, to assess general land quality with the other datasets, an overall measure was produced, combining soil metal concentrations into a single indicator of soil quality across the region. This was done using the log-transformed soil data for As, Cr, Ni, Pb and Se—the elements of potential concern to human health under the current UK CLEA guidelines (EA 2009). The key stages in developing this soil metal index were as follows:

1. For each metal, soil concentrations were classed into decile percentiles based on the cumulative distribution function (cdf) of the data and assigned a score from 1 to 10 such that higher concentrations were allocated a higher score.
2. Scores for the five metals were summed together to generate a total soil metal index for each soil sample point.
3. Mean total metal scores within each IGZ were then computed and presented in map format.

**Fig. 2:**
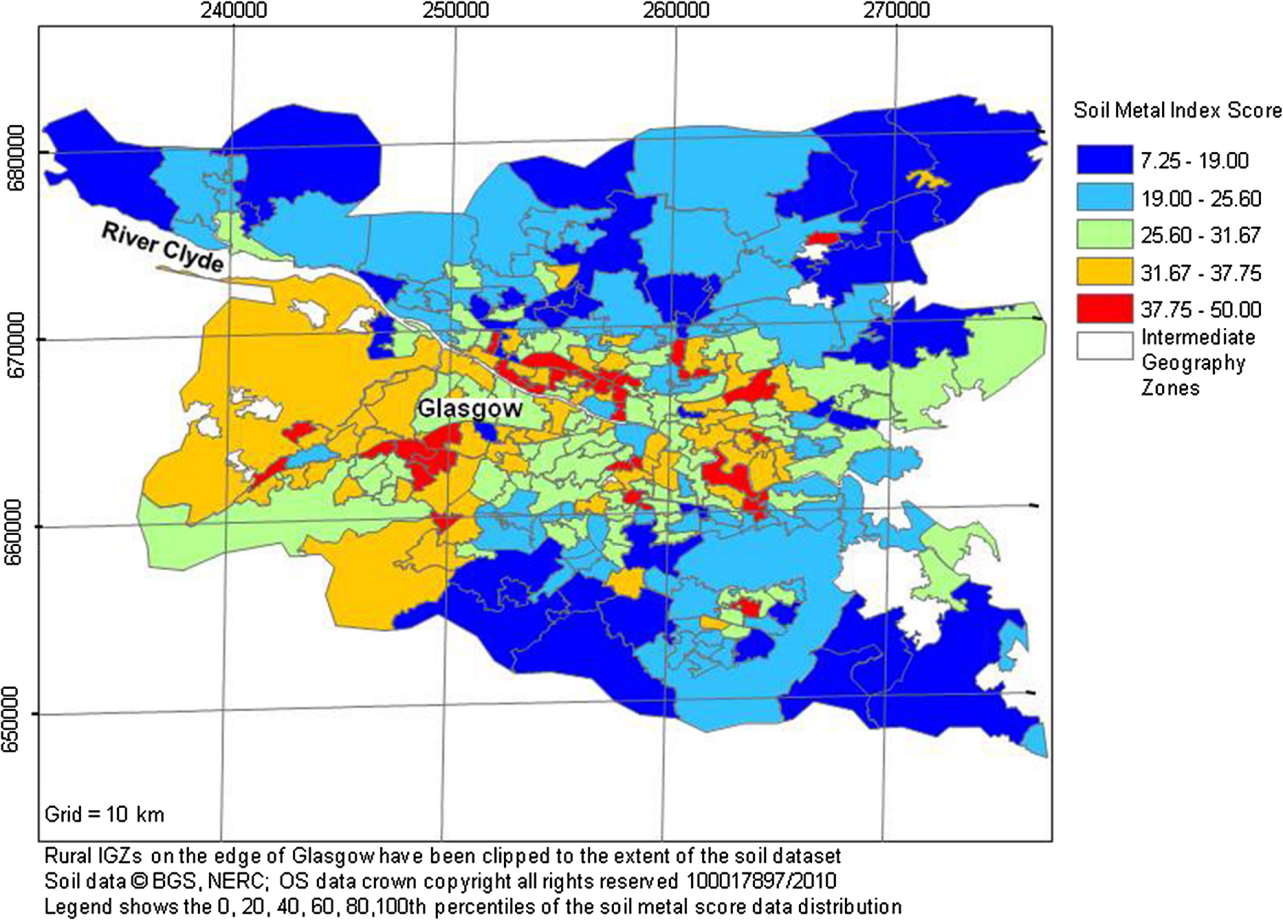
Map of combined soil metal index scores for each IGZ across the Glasgow study area (derived from Fordyce et al. 2012)

This relatively simple index was used as a summary measure of overall land quality; it does not indicate land likely to pose a threat to health (Fig. 2). Similar indices aggregating soil element concentrations to give a pollution index have been used in the analysis of soil data from other European cities (e.g. in Lithuania, Gregorauskiene et al. 2011).

### Air quality data

**Fig. 3:**
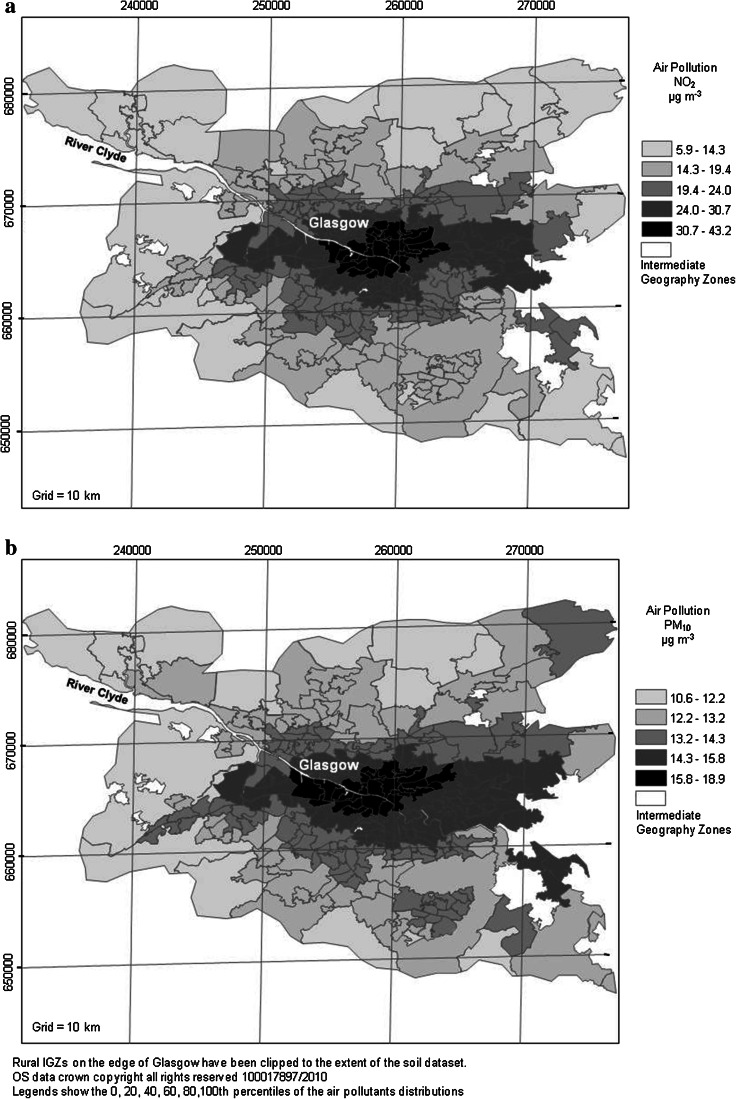
Mean **a** NO_2_ and **b** PM_10_ air pollution concentrations for each IGZ across the Glasgow study area (from SNS 2010)

Data on air quality consisted of ambient air pollution concentrations of nitrogen dioxide (NO_2_) and particulate matter (PM_10_). These parameters were selected as they are routinely monitored across Scotland. Modelled average (mean) pollution concentrations recorded at the IGZ level were used for the study. These were recorded as population-weighted means over the three-year period between 2002 and 2004 (Scottish Air Quality 2010) and were available from the Scottish Neighbourhood Statistics Website (SNS 2010). Modelled estimates were used since data and individual monitoring sites were not available on a small enough scale for this study. However, there was no uncertainty available on these measurements, so they were assumed to be known mean concentrations within the corresponding IGZs. Background concentrations were selected for the present study as they were recorded distant from potential sources, so were more representative of general air pollution levels in the city than kerbside or roadside locations (Fig. 3).

### Health data: respiratory cases

**Fig. 4:**
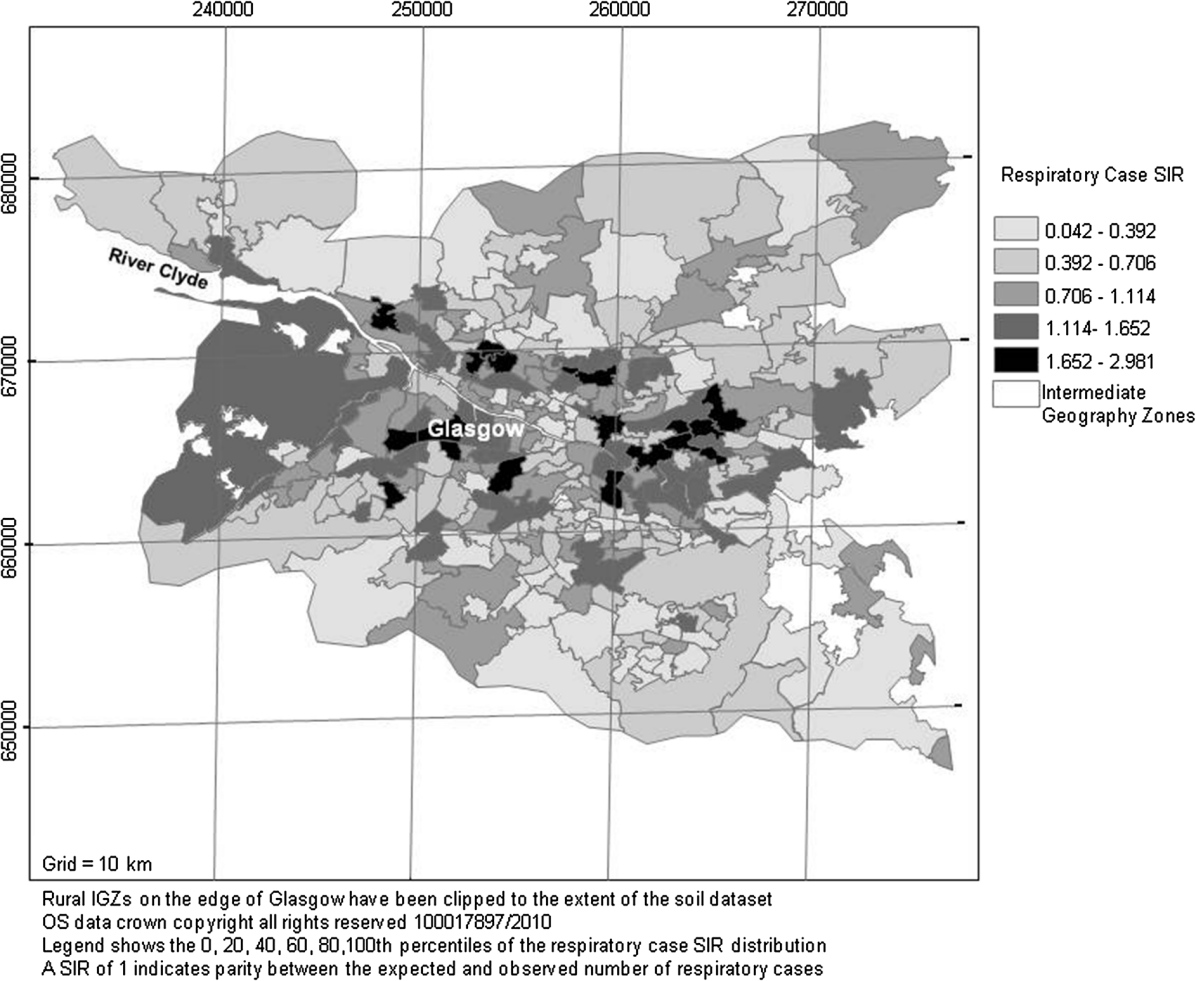
Map of respiratory case SIRs for each IGZ across the Glasgow study area (from SNS 2010)

Respiratory disease was chosen as the health indicator dataset selected for this study because it is one of the most common diseases in Scotland—therefore had substantial counts—and because it forms part of the Scottish Government health strategy (Scottish Government 2007). More importantly, respiratory disease is known to have biologically plausible links with the air pollutants and the soil contaminants selected for this study (DEFRA 2010; WHO 1996). The respiratory case data were available from the Information Services Division (ISD) of the National Health Service (NHS) of Scotland and were extracted from SNS (2010), with corresponding age- and sex-specific data available from ISD (2010). The data comprised the total number of hospital admissions in the year 2002 expressed as rates per 100,000 population recorded for each IGZ. Common methods of presenting health-based information include using the raw counts or the rate per head of population. However, these measures do not take into account the age and sex distribution of each IGZ, although it is widely accepted that there are differences in disease prevalence between the sexes and that older people and those who smoke are more likely to develop respiratory disease (ISD 2010). As a result, for the purposes of this study, standardised incidence ratios (SIRs) were generated to adjust for the population age and sex structure across Greater Glasgow (Morrison 2011) (Fig. 4). Standardised incidence ratios are commonly used in summarising health data and compare the observed versus expected number of cases in each IGZ based on age- and sex-specific rates from a standard population. A SIR of 1 indicates parity between the expected and observed number of cases (Sahai and Khurshid 1996). No information on smoking habit was available to take into account in this study, but deprivation has been used as a proxy indicator for smoking in similar studies (Lee et al. 2009).

### Deprivation data

**Fig. 5:**
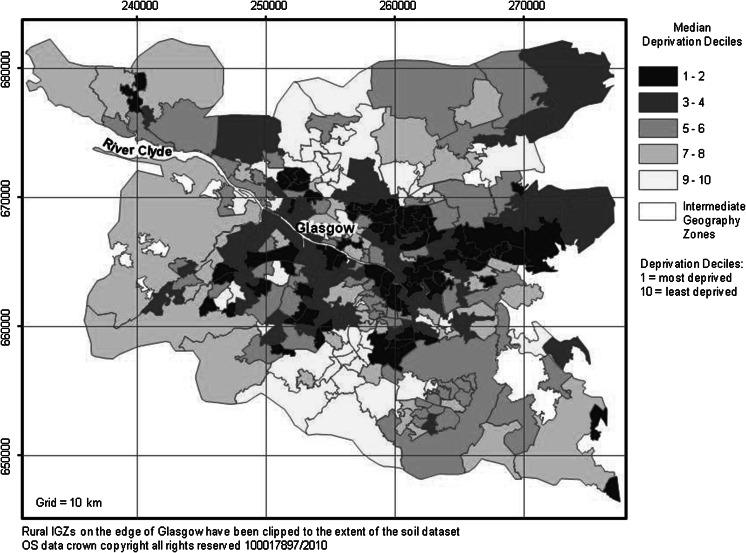
Median deprivation deciles for each IGZ across the Glasgow study area, based on reconstructed SIMD (SIMD 2010) excluding the health domain

Data on deprivation for this study were taken from the Scottish Index of Multiple Deprivation (SIMD 2010). It offers a relative measure of how deprived an area is for each data zone (DZ) in Scotland ranked from 1 (most deprived) to 6,505 (least deprived). The latest version of the SIMD was developed in 2009 using seven domains: health, education, employment, housing, income, access to services and crime (SIMD 2009). Since part of this project was to assess the relationships between health and deprivation across Glasgow, it was important to note that health was one of the domains included in the SIMD. Directly comparing these two variables would have produced erroneous conclusions, as health would have been accounted for in both datasets. Therefore, a new ranking system was constructed for the current project that excluded the health domain. The same methods and weighting scheme adopted by the SIMD (2009) were applied, but computing the ranks across six domains, minus health. As with the SIMD (2009), from this deprivation index of ranks, a classification of deciles was developed, whereby the ranks were grouped into ten equally distributed percentiles, by areas with similar deprivation characteristics. In this index, decile 1 represented the most deprived, whilst decile 10 corresponded to the least deprived DZ. Data zones represent smaller areas than IGZs; therefore, to convert the decile index from DZ level to IGZ level, the median value of the DZs within each IGZ was taken (Fig. 5).

### Statistical modelling and analysis

Initial assessments of the associations between the soil, air, respiratory case and deprivation datasets for the 279 IGZs across Glasgow were carried out using Pearson’s correlation coefficients, taking the 5 % significance level (*p* < 0.05) (Stockburger 2001). These were used to test the strength of linear association between any two variables, although for the respiratory case data, these did not take into account other potential confounding factors. The relationships with respiratory cases were then examined in more detail using generalised linear modelling (GLM). Normal linear modelling describes the expected value of the response variable as a linear combination of a set of explanatory variables (predictors) assuming a normal distribution. GLM is a generalisation of ordinary regression that—via a link function—allows the model to accommodate response variables that have non-normal distributions and/or that are nonlinearly related to the predictor variables (Wood 2006). GLM has been used in previous studies to assess associations between health indicators (response variable), air pollution and deprivation (predictors) (e.g. Lee et al. 2009). In the present study, GLM with a log-link function was used to model the SIRs for respiratory cases (Morrison 2011). When incorporating soil contaminants into the regression analysis, various approaches were explored. There were drawbacks to each approach. One possible method was to include all individual soil metal IGZ geometric means in the model. However, initial assessments of the soil metal data revealed that many were strongly correlated with each other (see next section and Table 2). This multi-collinear relationship caused problems when carrying out regression modelling. Multi-collinearity occurs when two or more variables are strongly correlated with one another and neither have a statistically significant impact on a regression model when the other is accounted for. To avoid multi-collinearity, soil metals were included in the model one at a time. Although this approach was adopted for the initial assessments, it overlooked a considerable amount of information for the other soil metals; hence, the results from models in which individual soil metals were included were treated with caution. As a second approach, the soil metal index for each IGZ was used to represent the land quality covariate in the model. Therefore, GLMs were produced separately to assess associations between the respiratory cases (response variable) and (1) individual soil contaminants and (2) the index of soil metals as a land quality indicator. In each case, the soil metal indicator was added as the first predictor variable, whilst deprivation and air quality were added subsequently to the GLMs. In each of the models, a standard 5 % significance level (*p* < 0.05) was considered. Model selection used Akaike’s information criterion (AIC), which measures the goodness of fit of a model based on the number of parameters included and the maximised likelihood for the model. The minimum AIC identified the ‘best-fit’ model (Wood 2006). All statistical analysis was carried out using the open source R code (CRAN 2009).

**Table 2:** Pearson’s correlation matrix of IGZ soil metal geometric mean concentrations

	As	Cr	Cu	Ni	Pb	Se	Zn
Cr	*0.214*						
Cu	*0.520*	*0.356*					
Ni	*0.524*	*0.477*	*0.777*				
Pb	*0.469*	*0.260*	*0.794*	*0.581*			
Se	*0.706*	*0.214*	*0.390*	*0.343*	*0.365*		
Zn	*0.393*	*0.377*	*0.797*	*0.611*	*0.758*	*0.286*	
K	*0.131*	−0.066	0.026	−0.010	−0.019	0.009	0.009

*n* = 279, *r* = 0.118 (*p* < 0.05) (Stockburger 2001). Figures shown in italics are statistically significant

## Results and discussion

### Spatial distributions

The natural and anthropogenic controls on the spatial distributions of soil metals across Glasgow have been described by Fordyce et al. (2012). This information and the IGZ maps of Glasgow prepared for the present study revealed that geometric mean soil metal concentrations were elevated in urban areas relative to rural areas (e.g. Ni, Fig. 1). The exception was K, which was included in the study as a control metal element. Concentrations of K were higher in soils in rural areas around Dumbarton and south-east of Glasgow due to the presence of sandstones and glacio-fluvial sand and gravel deposits (Fordyce et al. 2012; Morrison 2011). Higher individual soil metal concentrations and combined soil metal index scores were recorded in the south-west Glasgow—Paisley area; the shipbuilding centre in the River Clyde corridor to the west of the city centre and in the former industrial heartland in the east of the city (Figs. 1, 2). In the case of soil Ni, high values to the south-east of Glasgow around East Kilbride reflect the presence of basic volcanic bedrock in this area (Fig. 1) (Fordyce et al. 2012).

For air pollution, higher mean concentrations of both NO_2_ and PM_10_ were present in the city centre and urban areas of Glasgow than rural areas, as expected (Fig. 3). In terms of the health indicator, respiratory case SIRs were higher in eastern Glasgow but high incidences were also reported in the Paisley area as well as the south-west and north-west of the city (Fig. 4). The SIMD data revealed a partially similar pattern in that the most deprived areas were clustered in east, south-east and north-east Glasgow but several urban IGZs also had high levels of deprivation in the Paisley and Dumbarton areas. Several rural IGZs on the urban fringe were also classed as highly deprived due to the influence of the urban periphery on the IGZ classification (Fig. 5).

### Initial statistical assessments of environment versus health and deprivation indicators

As an initial assessment of the soil data, Pearson’s correlation coefficients between each of the IGZ geometric mean metal concentrations were computed (Table 2). Statistically significant (>0.118; *p* < 0.05) associations were observed between all soil metals, with the exception of K as expected. Potassium was included in the study as a control element as it is generally non-harmful and had a different spatial distribution from the other elements (Fordyce et al. 2012; Morrison 2011). The significant correlations between the other soil metals reflect the generally higher contents in urban versus rural soil and the fact that in urban soil impacted by pollution, several metal concentrations are elevated at the same location (Fordyce et al. 2012; Morrison 2011).

Relationships between the environment, health and deprivation datasets were also explored initially using Pearson’s correlation coefficients (Table 3). It should be noted that correlations with the deprivation index are negative as decile 1 represents the *most* deprived and decile 10 the *least* deprived areas. All variables showed a statistically significant correlation with one another. A very strong correlation (0.978; *p* < 0.05) was observed between air NO_2_ and PM_10_, as expected. Soil metal index score also showed significant correlations with air quality (0.392 NO_2_; 0.389 PM_10_; *p* < 0.05) indicating a spatial association between the two environmental media. Deprivation not only demonstrated significant correlations with air pollution (−0.286 NO_2_; −0.307 PM_10_; *p* < 0.05) but with soil metal index score (−0.213; *p* < 0.05) as well. The associations between deprivation and poor air quality were to be expected as they have been documented by previous studies (e.g. Fairburn et al. 2005).

**Table 3:** Pearson’s correlation matrix of IGZ soil metal index, respiratory case and deprivation variables

	Respiratory case SIR	Soil metal index	Deprivation	Air NO_2_
Soil metal index	*0.262*			
Deprivation	−*0.397*	−*0.213*		
Air NO_2_	*0.222*	*0.392*	−*0.286*	
Air PM_10_	*0.230*	*0.389*	−*0.307*	*0.978*

*n* = 279, *r* = 0.118 (*p* < 0.05) (Stockburger 2001). Figures shown in italics are statistically significant

However, the results for the soil metal index are important in the context of the environmental justice agenda as they demonstrate for the first time in the United Kingdom that chemical land quality is poorer in deprived areas across a city such as Glasgow. This relationship may reflect the fact that a substantial portion of the population still lives in former industrial areas in Glasgow such as the East End and the River Clyde corridor. Following clearance of former industrial sites, this poorer-quality land is often cheaper and more available for low-cost housing. Although heavy industry declined decades ago and some of these areas have been regenerated and redeveloped more than once, the legacy of high soil metal concentrations remains as metals have long residency times in soil. The results suggest that even today, the population in deprived areas is potentially at greater risk of exposure to higher soil metal concentrations than in other areas.

The initial results also showed a moderately significant correlation between deprivation and respiratory cases (−0.397; *p* < 0.05). Namely, respiratory case incidences were higher in areas of greater deprivation. Respiratory case SIRs also initially showed significant correlations (0.222–0.262; *p* < 0.05) with air pollution and soil metal index score (Table 3). However, these associations should be treated with caution since potential confounding factors were not taken into account in these preliminary univariate statistical comparisons. Therefore, the relationships between the respiratory cases and the environmental factors were explored further as follows.

### GLM of respiratory case rates versus soil metals, deprivation and air pollution

In order to identify associations between respiratory cases and possible causal environmental factors that were plausible rather than coincidental, GLM statistical assessments were carried out. Since the main interest in this study was to assess spatial relationships between a health indicator and land quality, the respiratory case SIRs response variables were modelled against soil metal content in the first instance before the other environmental variables were added as predictors in the models.

With the soil metal index as the only covariate in the model, a statistically significant relationship with respiratory case SIRs was observed initially (*p* < 0.0001) (Morrison 2011). This association remained significant (*p* = 0.0367) even when deprivation and air pollution were taken into account (Table 4). The least deprived deciles showed a statistically significant negative relationship with respiratory case SIRs in the model (*p* < 0.0448), namely the wealthier areas had lower rates of respiratory cases as expected. Surprisingly, the most deprived deciles showed no significant relationship with respiratory case incidence (Table 4). This may be because ‘averaging’ the deprivation classes for the IGZs from the more detailed DZ areas resulted in some low decile values for mixed rural–urban areas on the edge of Glasgow due to the influence of deprived areas on the urban periphery (Fig. 5). These largely rural areas on the urban fringe are not where most respiratory admissions are likely to occur. Respiratory case SIRs did not show a statistically significant relationship with air pollution (NO_2_ *p* = 0.0718, Table 4); however, air pollution was kept in the final model based on minimum AIC (Morrison 2011).

**Table 4:** GLM output of respiratory case SIRs against soil metal index, deprivation index deciles and air NO_2_

Coefficient	Estimate	SE	*p* value
Intercept	−0.6147	0.1956	*0.0019*
Soil metal index	0.0102	0.0048	*0.0367*
Decile 2	0.1069	0.1274	0.4022
Decile 3	0.1783	0.1242	0.1525
Decile 4	−0.0861	0.1628	0.5975
Decile 5	−0.1789	0.1465	0.2231
Decile 6	−0.3713	0.1841	*0.0448*
Decile 7	−0.4928	0.2140	*0.0221*
Decile 8	−0.5078	0.2100	*0.0163*
Decile 9	−0.4859	0.2014	*0.0165*
Decile 10	−0.5831	0.2580	*0.0246*
Air NO_2_	0.0099	0.0055	0.0718
AIC	381.62		

Deprivation Deciles: 1 = most deprived; 10 = least deprived. Figures shown in italics are statistically significant

*SE* Standard error

When modelling individual soil metal IGZ geometric means, soil Ni showed a significant relationship (*p* = 0.0056) with respiratory case SIRs even when deprivation and air NO_2_ were included (Table 5). These results are interesting because Ni is a known respiratory irritant (EA 2009) and Ni in air particulates has been linked to increased respiratory disease in previous studies (e.g. Patel et al. 2009).

**Table 5:** GLM output of respiratory case SIRs against soil Ni concentration, deprivation index deciles and air NO_2_

Coefficient	Estimate	SE	*p* value
Intercept	−0.5814	0.1785	*0.0013*
Soil Ni geometric mean	0.0031	0.0011	*0.0056*
Decile 2	0.1351	0.1264	0.2863
Decile 3	0.1841	0.1234	0.1368
Decile 4	−0.0517	0.161	0.7484
Decile 5	−0.2037	0.1496	0.1744
Decile 6	−0.3451	0.1838	0.0616
Decile 7	−0.4926	0.2148	*0.0226*
Decile 8	−0.4959	0.2113	*0.0197*
Decile 9	−0.4637	0.201	*0.0218*
Decile 10	−0.6089	0.2584	*0.0192*
Air NO_2_	0.0138	0.0053	*0.0101*
AIC	380.96		

Deprivation deciles: 1 = most deprived; 10 = least deprived. Figures shown in italics are statistically significant

*SE* Standard error

However, it should be emphasised that no causal links between respiratory cases and soil metal content are implied by these results as information on exposure linkages between soil and the population in Glasgow is lacking. On the one hand, the results may simply reflect the fact that soil metal concentrations and respiratory disease incidence tend to be higher in urban areas compared to the rural periphery of the city. On the other hand, the results may indicate an association between soil and air quality. This study has shown spatial concurrence between poor air quality and soil chemical quality, with higher soil metal and air pollution concentrations in the urban versus the rural environment around Glasgow. The links between air particulates and respiratory disease have been well established (DEFRA 2010; Dockery and Pope 1994), and one of the main concerns in terms of increased health risk is exposure to metals such as Cr and Ni in PM_10_ air particulates as these have been shown to cause pulmonary damage and increased cases of respiratory disease (Bell 2012; Costa and Dreher 1997). It is interesting to consider the contribution that soil makes to airborne particulate material and to concentrations of metals in air. Several studies have demonstrated the importance of re-suspended soil material to air particulate matter. Young et al. (2002) found that during summer–autumn months, 74 % of airborne PM_10_ particulates were derived from soil in Bakersfield, California. Wells et al. (2007), Laidlaw and Filippelli (2008) and Laidlaw et al. (2012) demonstrated similar results in cities across the United States (US) with a seasonal correlation between soil re-suspension and air Pb concentrations. Recent work by Cave and Chenery (2010) in the United Kingdom also suggests that perhaps 45 % of the PM_10_ particulates in air may be soil-derived. Furthermore, Laidlaw and Filippelli (2008) and Zahran et al. (2013) found clear links between the re-suspension of soil particles, concentrations of Pb in air and seasonal variations in child blood lead levels in several US cities demonstrating a soil–air particulate–child exposure pathway. In another recent study, Broadway et al. (2010) showed that the majority of Cr present in Glasgow soils was in the CrIII form, which is generally considered essential for human health. However, CrVI—a known respiratory irritant and carcinogen—was present in soils impacted by waste from the former chromate ore processing plant located in the south-east of the city. Laboratory-based tests to simulate soil particulate inhalation demonstrated that soil CrVI was bioaccessible. Therefore, in the context of the environmental justice agenda, it cannot be ruled out that exposure to soil metals, via inhalation of windblown, airborne and household dust particles, adds to the metal loading of populations in Glasgow and that this exposure is likely to be greater in more deprived areas due to the legacy of soil pollution in the city. However, further investigations would be required to assess soil–air–population relationships more closely.

## Conclusions

This study examined the relationships between respiratory cases and soil and air quality and deprivation in IGZs across Glasgow. Under the environmental justice agenda, the links between deprivation and poor air and water quality, derelict land and lack of access to green space have already been established in the United Kingdom but the chemical quality of land has not been considered until now. This study has demonstrated for the first time that there is a spatial association between deprivation and poor soil chemical quality for a major post-industrial UK city. Even decades after heavy industry ceased, the legacy of the city’s industrial past remains and soil metal concentrations are higher in the more deprived areas of Glasgow. The results suggest that the population in the more deprived areas of Glasgow is potentially exposed to higher soil metal concentrations than in other areas of the city.

This study has also shown that soil metal content indicates a statistically significant association with respiratory case incidence across the city even when deprivation and air pollution are taken into account. It should be stressed that no causal links between soil metal content and respiratory disease are implied by this study but the results are interesting given the contribution soil metals may make to air particulates and the known associations between air pollution and health. Therefore, the relationships highlighted in this study warrant further investigation.

None of the commonly used measures of deprivation currently include an environmental factor. For example, the SIMD is an index based on a number of social and health indicators, but does not have an environmental component. Although links between soil chemical quality and health outcomes are difficult to assess, it is nonetheless recommended that in order to improve the environment and the quality of life in deprived areas, chemical land quality should be taken into account in addition to indicators of air, water and green space quality in deprivation and environmental justice assessments in the future.
